# A novel mutation in the N-terminal domain of *Drosophila* BubR1 affects the spindle assembly checkpoint function of BubR1

**DOI:** 10.1242/bio.021196

**Published:** 2016-10-14

**Authors:** Marie Duranteau, Jean-Jacques Montagne, Zohra Rahmani

**Affiliations:** CNRS, Institut Jacques Monod, UMR7592, Université Paris Diderot, Paris Cedex 13 75205, France

**Keywords:** Mitosis, Kinetochore, Spindle assembly checkpoint, Cdc20

## Abstract

The spindle assembly checkpoint (SAC) is a surveillance mechanism that ensures accurate segregation of chromosomes into two daughter cells. BubR1, a key component of the SAC, also plays a role in the mitotic timing since depletion of BubR1 leads to accelerated mitosis. We previously found that mutation of the KEN1-box domain of *Drosophila* BubR1 (*bubR1-KEN1* mutant) affects the binding of BubR1 to Cdc20, the activating co-factor of the APC/C, and does not accelerate the mitotic timing despite resulting in a defective SAC, which was unlike what was reported in mammalian cells. Here, we show that a mutation in a novel *Drosophila* short sequence (*bubR1-KAN* mutant) leads to an accelerated mitotic timing as well as SAC failure. Moreover, our data indicate that the level of Fzy, the *Drosophila* homolog of Cdc20, recruited to kinetochores is diminished in *bubR1-KEN1* mutant cells and further diminished in *bubR1-KAN* mutant cells. Altogether, our data show that this newly identified *Drosophila* BubR1 KAN motif is required for a functional SAC and suggest that it may play an important role on Cdc20/Fzy kinetochore recruitment.

## Abstract (precis)

**Summary:** We have identified a novel short motif sequence in the *Drosophila* BubR1 protein which when mutated affects the amount of Cdc20 recruited to the kinetochores and leads to an abrogation of the SAC.

## INTRODUCTION

The mitotic spindle assembly checkpoint (SAC) monitors proper bipolar attachment of chromosomes via their kinetochore to the mitotic spindle. BubR1 and Mad2 are two key proteins essential for the SAC-mediated inhibition of the anaphase promoting complex or cyclosome (APC/C), an E3 ubiquitin ligase that targets Cyclin B and Securin for degradation and promotes anaphase entry. BubR1 mediates APC/C inhibition by binding to Cdc20, the activating co-factor of the APC/C, via two conserved KEN motifs (Hardwick et al., 2000; Burton and Solomon, 2007; King et al., 2007; Musacchio and Salmon, 2007; Sczaniecka et al., 2008; Kulukian et al., 2009).

BubR1, like Mad2, was previously shown to contribute to the timing of mitosis since depletion of Mad2 and BubR1 in mammalian cells resulted not only in SAC failure but also in a strong acceleration of the time elapsing between nuclear envelope breakdown and anaphase onset compared to control cells (Meraldi et al., 2004). Several studies have shown that the mutation or deletion of either KEN domain abolishes the SAC and leads to a shorter mitotic timing when compared to wild-type cells (WT) (Malureanu et al., 2009; Elowe et al., 2010; Lara-Gonzalez et al., 2011); however, we previously reported that *Drosophila* larval neuroblasts expressing a mutated BubR1 KEN1-box mutated for BubR1 (*bubR1-KEN* mutant renamed bubR1-KEN1 herein) do not have an accelerated mitotic timing despite the fact that the SAC function is abolished (Rahmani et al., 2009), thereby suggesting that either a second KEN-motif or an unknown motif could contribute to the mitotic timing function of BubR1. More recently, inhibition of Mps1 in mammalian cells was also shown to lead to an accelerated anaphase onset (Hewitt et al., 2010; Maciejowski et al., 2010; Santaguida et al., 2010), and mitosis was also reported to be accelerated in retinal pigment epithelium (RPE) cells fully depleted for Mad1 (Rodriguez-Bravo et al., 2014); however, this is not the case in *Drosophila* where *mad1* null mutant cells display a normal mitotic timing similar to WT cells (Emre et al., 2011). Therefore, the previously referred timer and checkpoint functions originally defined as being potentially distinct functions are more likely to reflect a different degree of depletion of the SAC proteins rather than being separate entities. Here, we show that the SAC is abolished and that the mitotic timing is accelerated in *Drosophila* larval neuroblasts expressing a novel mutated BubR1 sequence (*bubR1-KAN* mutant), and we provide evidence that this novel *Drosophila* BubR1 KAN motif may play an important role on the level of Fzy/Cdc20 recruited to kinetochores.

## RESULTS AND DISCUSSION

**Fig. 1.**
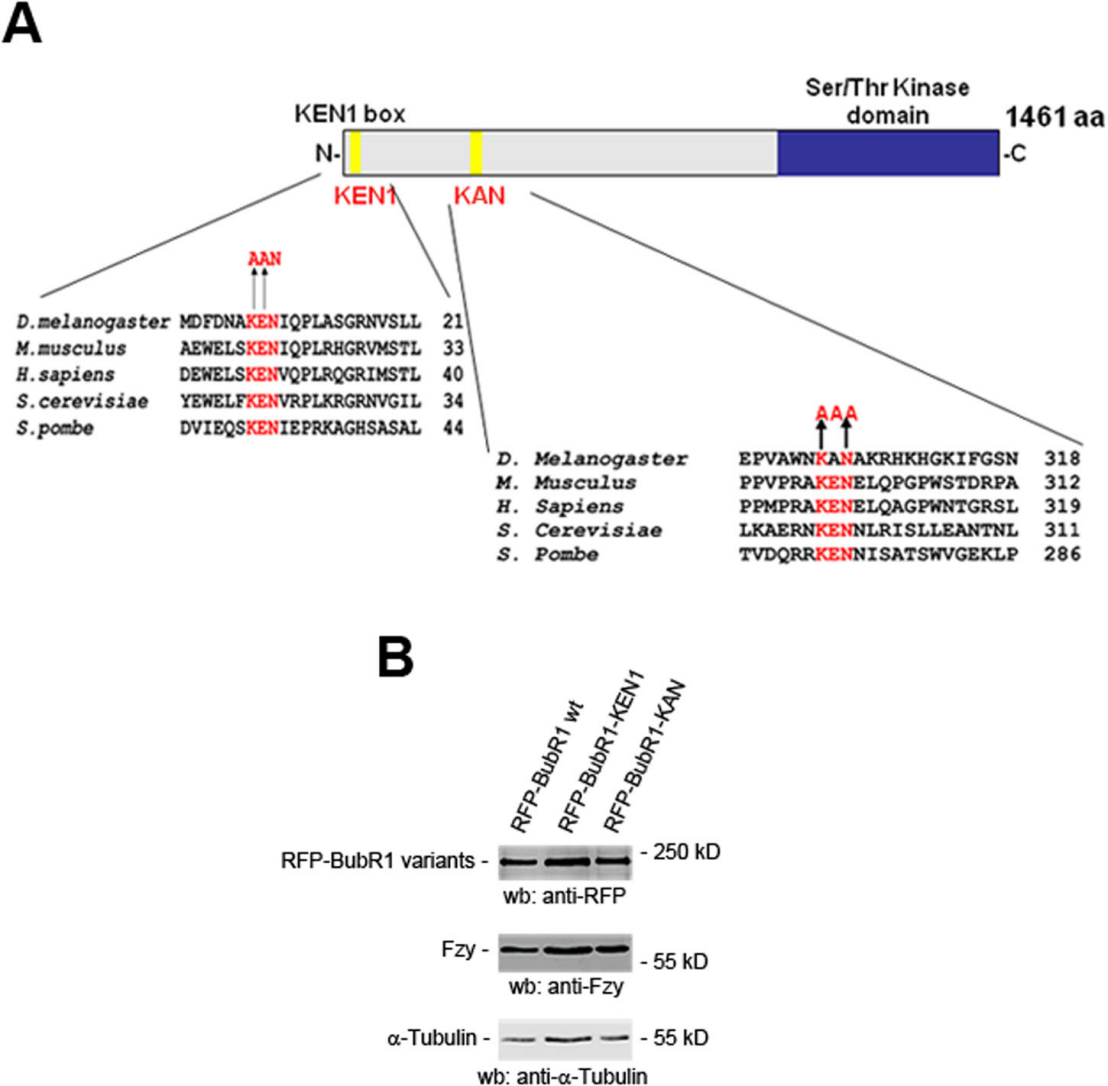
**Structure of BubR1-KEN1 and BubR1-KAN mutations and expression levels of the various BubR1 WT, BubR1-KEN1 and BubR1-KAN mutant transgenes.** (A) Sequence alignments of the KEN1 box (left) and the KEN2 or KAN box (right) in *Drosophila melanogaster*, *Mus musculus*, *Homo sapiens*, *Saccharomyces Cerevisiae*, and *Saccharomyces Pombe*. Residues altered in *bubR1-KEN1* and in *bubR1-KAN* mutants are indicated in red. (B) Western blot showing equivalent expression levels of homozygous transgenic lines expressing RFP-BubR1 WT, RFP-BubR1-KEN1 and RFP-BubR1-KAN in the *bubR1^1^* null mutant background. Extracts of dissected larval brains were normalized for total protein before loading of the gel. The same blot was stripped and reprobed with tubulin antibody to verify that equal amount of protein extracts were loaded on each lane.

The KEN1-box domain is evolutionarily well-conserved among all studied species, including *Drosophila*, whereas the KEN2-box domain does not seem to be (Fig. 1A). However, sequence alignment of the human KEN2 motif with several species including *Drosophila* pointed to a novel short linear motif that consisted of lysine, alanine, and asparagine residues (KAN) (Fig. 1A). The crystal structure of Cdc20 bound to the first KEN1-box of BubR1 was reported and showed that the acidic chain of the glutamate residue interacts directly with Cdc20, and that the side chain of the lysine residue forms hydrogen bonds with two asparagine residues of Cdc20 (Chao et al., 2012; Tian et al., 2012). Therefore, the presence of an alanine residue instead of a glutamate residue makes it unlikely that the KAN motif corresponds to a canonical KEN domain. Interestingly, Vleugel et al. reported an alternative sequence alignment of the BubR1 protein based on the published genome of 60 eukaryotes species in which three residues (Q, E, N) of the *Drosophila* BubR1 sequence were aligned with the mammalian KEN2 motif (Vleugel et al., 2012). Whether the QEN short motif may constitute an alternative possible second KEN domain in *Drosophila* remains to be determined. Despite this observation, we generated a mutant allele of the putative KAN sequence of *Drosophila* BubR1 by replacing Lys303 and Asn305 (KAN) by two alanines (AAA) in a previously characterized and fully functional mRFP-BubR1 construct (Buffin et al., 2005). Several transgenic lines were established, and they were all capable of rescuing the lethality observed with the *bubR1^1^* null mutation, similar to that reported for the *bubR1-KEN* mutation (Rahmani et al., 2009). Moreover, the RFP-BubR1 WT, RFP-BubR1-KEN1, and RFP-BubR1-KAN transgenes (expressed in a *bubR1^1^* homozygous null genetic background) showed similar levels of expression (Fig. 1B). These flies of genotype *bubR1^1^; P[w+, mRFP-bubR1-KAN]* will be called hereafter *bubR1-KAN* mutant flies. We then tested the functionality of the SAC in *bubR1-KAN* mutant cells by treating WT or *bubR1-KAN* mutant cells with colchicine, a microtubule depolymerizing agent. We found that, unlike WT neuroblasts for which the mitotic density rose threefold, *bubR1-KAN* neuroblasts failed to arrest in mitosis after 1 h colchicine treatment (Table S1) thereby indicating that *bubR1-KAN* mutant neuroblasts were checkpoint defective. The fact that the RFP-BubR1-KAN transgene rescues in a single copy the lethality of *bubR1^1^* mutant homozygotes (Table S1) indicates that the transgene is fully functional and argues against the possibility that the defective SAC observed in *bubR1-KAN* mutant neuroblasts may be due to a disruption of the BubR1 structure or its stability.

**Fig. 2.**
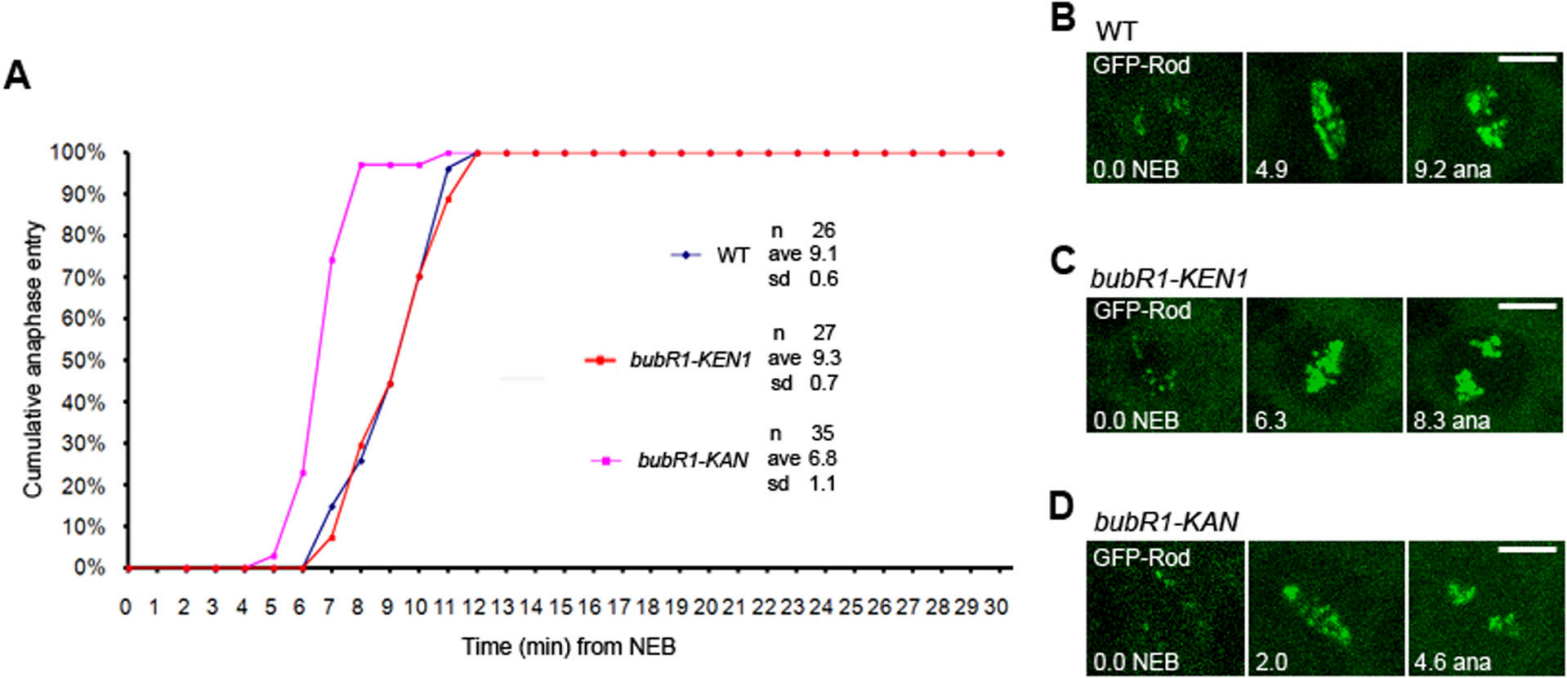
**Mitotic timing in *bubR1-KEN1*, *bubR1-KAN* mutant neuroblasts.** (A) Comparative mitotic timing of WT, *bubR1-KEN1* and *bubR1-KAN* mutant cells. NEB is defined as when GFP-Rod begins to be visible on kinetochores. *bubR1-KEN1* cells (red squares) show no change in mitotic timing relative to WT cells (9.3 min vs 9.1 min, *P*>0.5). *bubR1-KAN* cells (pink squares) enter anaphase earlier than WT cells (blue diamonds) (6.8 min vs 9.1 min, *P*<0.005). (B-D) Still frames extracted from typical movies used for the determination of the mitotic timing (from NEB to anaphase). See also Movies 1, 2, 3. *n*, number of cells analyzed; s.d., standard deviation. Scale bars: 5 µm.

We previously found that, unlike what was reported in mammalian cells (Meraldi et al., 2004; Malureanu et al., 2009; Elowe et al., 2010; Lara-Gonzalez et al., 2011), mutation of the KEN1-box domain of *Drosophila* BubR1 did not accelerate the mitotic timing despite resulting in a defective SAC (Rahmani et al., 2009). Therefore, we looked at WT, *bubR1-KEN1* and *bubR1-KAN* mutant cells by live imaging to measure the mitotic timing. For this, we followed kinetochores by employing GFP-tagged Rod, a kinetochore protein that is part of the RZZ complex (Buffin et al., 2005). Whereas WT neuroblasts spent an average of 9.1±0.6 min (Fig. 2A,B; Movie 1) between nuclear envelope breakdown (NEB) and the anaphase onset, *bubR1-KAN* mutant neuroblasts showed an accelerated mitotic timing with an average of 6.8±1.1 min (Fig. 2A,C; Movie 3). *bubR1-KEN1* mutant neuroblasts showed no change in mitotic timing, as previously reported in *Drosophila* (9.3±0.7 min, Fig. 2A,D; Movie 2). These observations suggest that the KEN domain and the KAN sequence may have distinct effects on the *Drosophila* BubR1-mediated mitotic timing.

Mad2 and BubR1, along with Bub3, inhibit the APC/C by binding Cdc20, a cofactor of the APC/C. This assembly is called the mitotic checkpoint complex (MCC) and it inhibits the activity of the APC/C towards certain key substrates required for anaphase onset (Sudakin et al., 2001; Tang et al., 2001). Meraldi et al. previously showed that the maintenance of a minimum mitotic timing interval in HeLa cells was dependent on Mad2 and BubR1 (Meraldi et al., 2004). Interestingly, Li et al. showed that the recruitment of Cdc20 to the kinetochores in *Drosophila* neuroblasts is dependent only on BubR1 but not Mad2 (Li et al., 2010). Therefore, we asked if *bubR1-KEN1* or *bubR1-KAN* mutations were affecting the BubR1-mediated recruitment of Cdc20 to the kinetochores.

**Fig. 3.**
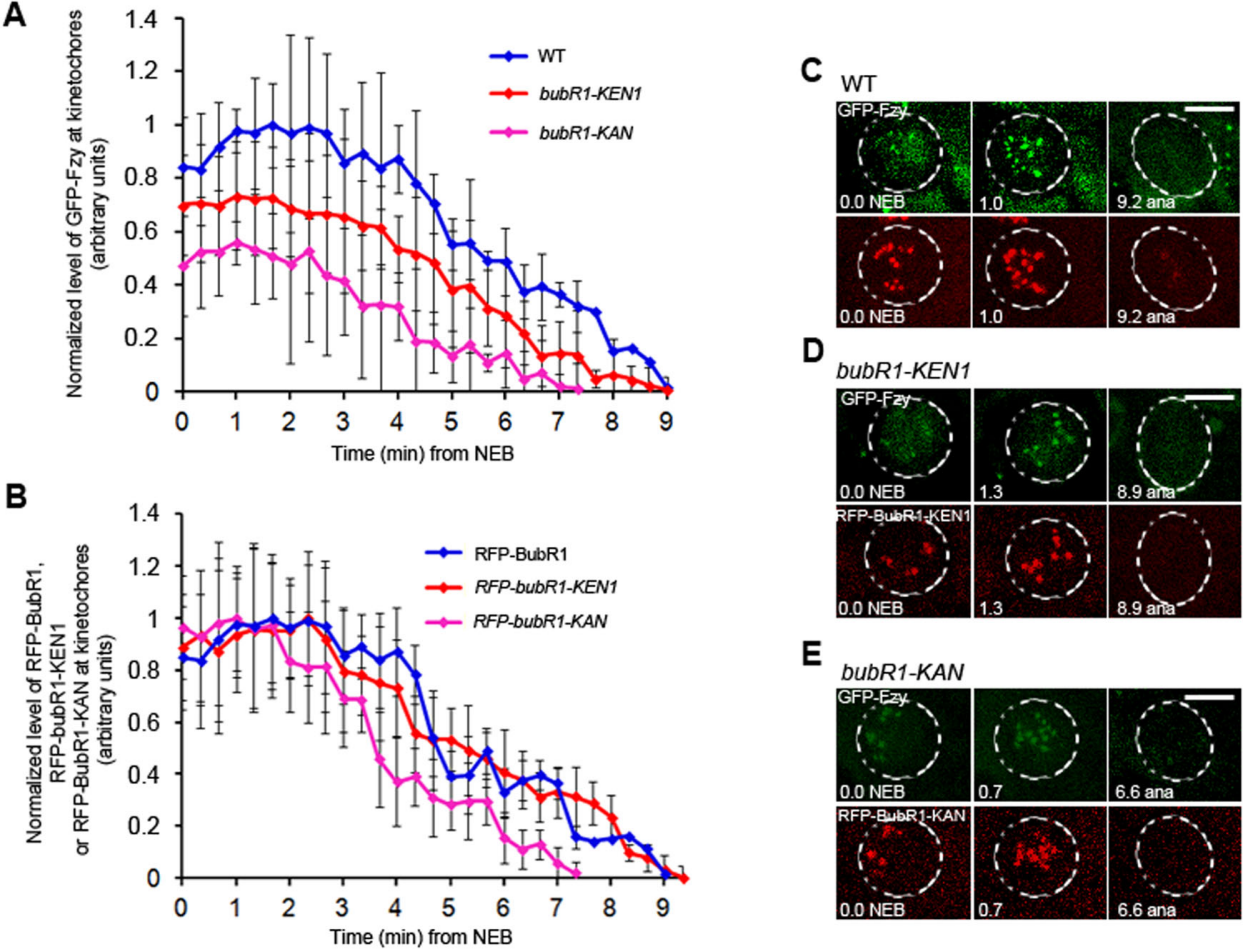
**Quantitative analysis of GFP-Fzy signal at kinetochores in BubR1 WT, *bubR1-KEN1* and *bubR1-KAN* mutant neuroblasts.** (A) The level of GFP-Fzy signal recruited at the kinetochores is lower in *bubR1-KEN1* (red diamonds), and even further reduced in *bubR1-KAN* (pink squares) mutant neuroblasts compared to the WT cells (blue squares). Values represent the mean and the s.e.m. (error bars) derived from approximately 40 kinetochores from 8 cells. (B) The level of RFP-BubR1 WT (blue diamonds), RFP-BubR1-KEN1 (red diamonds) and RFP-BubR1-KAN (pink diamonds) signal recruited at kinetochores is similar. (C-E) Still frames taken from representative movies used for the quantification of GFP-Fzy as well as for the quantification of RFP-BubR1 WT, RFP-BubR1-KEN1 and RFP-BubR1-KAN in WT, *bubR1-KEN1 and bubR1-KAN* mutant cells. See also Movies 4, 5, 6. *n*, number of cells analyzed. Scale bars: 5 µm.

We measured quantitatively the kinetochore recruitment of GFP-tagged Fzy in WT, *bubR1-KEN1*, and *bubR1-KAN* mutant cells. In WT neuroblasts, GFP-Fzy was at first cytoplasmic and then entered the nucleus at NEB. Right after the NEB, GFP-Fzy signal appeared on kinetochores and stayed visible until anaphase onset (Fig. 3C; Movie 4). GFP-Fzy behaved similarly in *bubR1-KEN1*, and *bubR1-KAN* mutant cells (Fig. 3D,E; Movies 5 and 6); however, the level of GFP-Fzy recruited to the kinetochores right after NEB was lower in *bubR1-KEN1* mutant cells than in WT cells, and reduced even further in *bubR1-KAN* mutant cells (Fig. 3A). Therefore, both BubR1 KEN1 and BubR1 KAN mutations do seem to have an influence on the recruitment of Fzy to kinetochores.

Recruitment of Cdc20 to kinetochores in HeLa cells was previously shown to be dependent on both BubR1 and Mad2, and deletion of the BubR1 KEN1 domain abrogated the interaction with Cdc20 and Mad2 whereas deletion of the BubR1 KEN2 domain had no effect on the interaction (Lara-Gonzalez et al., 2011). In contrast, kinetochore recruitment of Cdc20/Fzy in *Drosophila* neuroblasts was reported to be independent of Mad2 and to require only BubR1 (Li et al., 2010). Therefore, the lower level of GFP-Fzy recruited in *bubR1-KAN* and *bubR1-*KEN1 mutant cells versus BubR1 WT cells may be due to a difference in the kinetochores recruitment levels of BubR1-KAN and BubR1-KEN1 compared to BubR1-WT. To examine this possibility, we measured quantitatively the recruitment of these two mutant proteins to kinetochores as well as the WT protein during mitosis by expressing RFP-tagged forms of BubR1 WT, BubR1-KEN1 or BubR1-KAN in the *bubR1^1^* null genetic background. Our results showed that they are all recruited to the kinetochores to the same level and the levels followed the same profile over time (Fig. 3B). Moreover, the protein expression level of the RFP-BubR1 WT, RFP-BubR1-KEN1 and RFP-BubR1-KAN is equivalent (Fig. 1B). Therefore, the difference in GFP-Fzy recruitment at the kinetochores is not due to different levels of BubR1-KAN or BubR1-KEN1 vs BubR1 WT at kinetochores. Moreover, our findings indicate that in *bubR1-KEN1* mutant cells (in which the KAN sequence is functional) higher levels of Fzy are recruited to the kinetochores compared to the *bubR1-KAN* mutant cells (in which the KEN1 motif is functional). Therefore, these data suggest that the *Drosophila* BubR1 KAN motif may have an important role in Cdc20/Fzy kinetochore recruitment. Interestingly, several studies reported the identification of additional conserved motifs in human BubR1: an internal Cdc20 binding domain (IC20BD), also named Phe-box (for its phenylalanine containing region), a D-box located downstream of the Phe-box and the ABBA motif which encompasses the Phe-box (Lischetti et al., 2014; Di Fiore et al., 2015; Diaz-Martinez et al., 2015). These motifs were shown to contribute to Cdc20 binding and its recruitment to kinetochores (Lischetti et al., 2014; Di Fiore et al., 2015), and, although they were not essential for the SAC per se, they appeared to make the SAC more efficient (Lischetti et al., 2014; Di Fiore et al., 2015; Diaz-Martinez et al., 2015). Similarly, the *Drosophila* BubR1 KAN motif is also important for the recruitment of Cdc20/Fzy to kinetochores. However, unlike the IC20BD/Phe-box/ABBA motif, the *Drosophila* BubR1 KAN motif is required for the SAC.

**Fig. 4.**
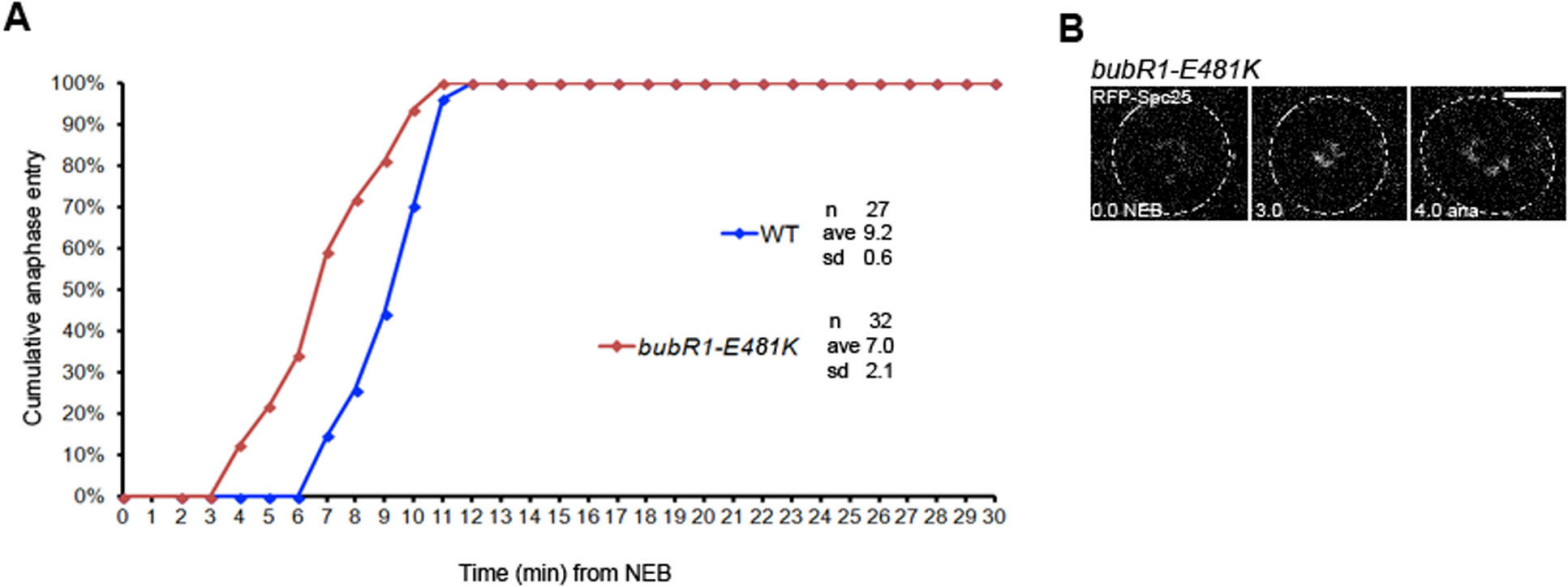
**Mitotic timing in *bubR1-E481K* mutant neuroblasts.** (A) Comparative mitotic timing of WT and *bubR1-E481K* mutant cells. NEB is defined as when RFP-Spc25 begins to be visible on kinetochores. *bubR1-E481K* mutant cells (brown diamonds) enter anaphase earlier than WT cells (blue diamonds) (7 min vs 9.2 min, *P*<0.00002). (B) Still frames extracted from typical movies used for the determination of the mitotic timing (from NEB to anaphase). See also Movie 7. *n*, number of cells analyzed; s.d., standard deviation. Scale bar: 5 µm.

Previous studies have shown that depletion of Mad1, Mad2, BubR1, or Mps1 result in an acceleration of mitosis, thereby indicating that these proteins specify the duration of the mitotic timing (Meraldi et al., 2004; Hewitt et al., 2010; Maciejowski et al., 2010; Santaguida et al., 2010; Rodriguez-Bravo et al., 2014). These proteins can also inhibit the APC/C independently of their kinetochore localization by assembling a cytosolic MCC (Kulukian et al., 2009; Malureanu et al., 2009). Our data suggest that the duration of the mitotic timing in *Drosophila* is correlated with the amount of Cdc20/Fzy recruited to kinetochores and that a new *Drosophila* KAN short motif may act by recruiting additional Cdc20/Fzy protein. However, these data cannot exclude the possibility that the pool of MCC required for a normal mitotic timing can still be assembled in the cytoplasm even if the *bubR1-KAN* mutant retains its kinetochore association. If this is the case, then preventing BubR1 from being recruited to kinetochores should not affect the assembly of the cytoplasmic pool of MCC required for a normal mitotic timing, and consequently cells should not have an accelerated mitotis. Therefore, in order to address this possibility, we prevented BubR1 association to kinetochores by generating a mutation in the glutamate 481 (referred to as *bubR1-E481K* mutant and homologous to E406 in human) of the Bub3 binding region of BubR1. This conserved residue was previously shown to be essential for the interaction of BubR1 with Bub3 and for the recruitment of BubR1 to kinetochores (Harris et al., 2005; Larsen et al., 2007; Derive et al., 2015). As reported by others in mammalian cells and in *Drosophila*, the mutation abolished kinetochore recruitment of the BubR1-E481*K* construct (data not shown). We then looked at WT and *bubR1-E481K* mutant cells by live imaging to measure the mitotic timing. For this, we used RFP-tagged Spc25, a kinetochore protein that is part of the Ndc80 complex, to follow kinetochores. Whereas WT neuroblasts spent an average of 9.2±0.6 min (Fig. 4A) between the NEB and the anaphase onset, *bubR1-E481K* mutant neuroblasts showed an accelerated mitotic timing with an average of 7±2.1 min (Fig. 4A,B; Movie 7). These data indicate that when BubR1 cannot be recruited to kinetochores, the mitotic timing is accelerated and supports our observation that the duration of mitotic timing in *Drosophila* is correlated with the amount of Cdc20/Fzy recruited to kinetochores. Interestingly, it was recently shown that the MCC can bind a second Cdc20 that is already bound to the APC/C in HeLa cells and that this binding is mediated by the BubR1 KEN2 box (Izawa and Pines, 2015). Thus, even though the BubR1 KEN1 box is essential to form the core MCC that prevents the activation of the APC/C, these observations argue that the BubR1 KEN2 box is necessary to rapidly inhibit the pool of Cdc20 already bound to the APC/C (Izawa and Pines, 2015). Our observation that the level of Cdc20/Fzy recruited to kinetochores is diminished in *bubR1-KAN* mutant cells compared to *bubR1-KEN* mutant and WT cells suggests that the *bubR1-KAN* mutant may recruit Cdc20/Fzy less efficiently to kinetochores. However, we cannot presently exclude the possibility that the accelerated timing observed in the *bubR1-KAN* mutant could also be due to its inability to inhibit the pool of Cdc20/Fzy already bound to the active APC/C. Altogether, our data show that this newly identified *Drosophila* BubR1 KAN motif is required for a functional SAC and suggest that it may play an important role on Cdc20/Fzy kinetochore recruitment.

## MATERIALS AND METHODS

### Fly stocks

GFP-Rod, RFP-Spc25, *bubR1-KEN* and *bubR1^1^* mutants have been described previously (Basu et al., 1999; Logarinho et al., 2004; Buffin et al., 2005, 2007; Schittenhelm et al., 2007; Rahmani et al., 2009). GFP-Fzy transgenic line was obtained from the laboratory of Jordan Raff (University of Oxford, Oxford, UK) and described previously (Raff et al., 2002).

### Cytology

Third instar larval brains were dissected in 0.7% NaCl and directly fixed and stained in aceto-orcein as previously described (Williams et al., 2003). The mitotic density (average number of mitotic cells per microscopic field) in response to colchicine-induced depolymerization of microtubules and the aneuploidy rates (Table S1) were determined as previously described (Rahmani et al., 2009). Cells were observed by phase-contrast microscopy using a Nikon Microphot microscope and a Zeiss 63X phase contrast objective.

### *In vivo* observation of larval neuroblasts and quantitative immunofluorescence

Wild-type, *bubR1-KEN1*, *bubR1-KAN*, *bubR1-E481K* wandering third instar larvae were dissected in Shields and Sang M3 insect medium (Sigma) supplemented with 10% fetal bovine serum (FBS) and penicillin-streptomycin. Three to four larval brains were immediately transferred into 15 µl M3 medium supplemented with 10% FBS, penicillin-streptomycin, 10 µg/ml insulin and 5 µg/ml of fly extract. Brains were mounted on a standard membrane (Yellow Springs Instruments, Yellow Springs, Ohio) and placed on a stainless steel slide as previously described (Kiehart et al., 1994).

Brains were imaged with a Perkin Elmer Ultraview spinning disk confocal head mounted on a Zeiss Axiovert microscope with a 100× (N.A. 1.4) lens and a 1× binning. At 20-s intervals, Z-series consisting of no more than seven 1-µm steps were acquired. Time-lapse image series were converted into movies using Metamorph (Universal Imaging) and ImageJ software and were processed using Adobe Photoshop. All movies frames are maximum intensity projections. GFP-Rod (or RFP-Spc25) signal was used to monitor the mitotic timing between NEB and anaphase onset. Nuclear envelope breakdown (NEB) was defined as when the GFP-Rod (or RFP-Spc25) signal began to be visible on kinetochores. Anaphase onset was defined as the moment sister kinetochores (marked with the GFP-Rod or the RFP-Spc25 signal) began to separate. In some movies (Fig. 3D,E), we followed RFP-BubR1, in which case NEB was defined as the moment RFP signal on kinetochores begins to rapidly intensify [since some signal is present even in prophase (Buffin et al., 2005)]. The level of GFP-Fzy recruited at kinetochores was quantitatively measured by expressing one copy of GFP-Fzy in WT, *bubR1-KEN1*, or *bubR1-KAN* neuroblasts. Films were processed as above and ImageJ was used for image quantification. Kinetochore fluorescence was quantified using the mean intensity of circle area for each kinetochore after background subtraction and bleaching correction, and normalized to the mean fluorescence intensity of the nearby cytoplasm for each time point. Values represent the mean and the s.e.m. (error bars) derived from approximately 40 kinetochores from 8 cells. Similar treatment was performed for measuring the level of RFP-BubR1-KEN1 and RFP-BubR1-KAN signals at kinetochores (Fig. 3B).

### Construction of bubR1-KAN and bubR1-E481K

The *Drosophila* N-terminal KAN sequence (Lys303 Ala304 Asn305) was mutated into Ala303 Ala304 Ala305 (AAA) by using a QuikChange II XL Site-directed Mutagenesis Kit (Stratagene) and the following primers: 5’-CCGGTGGCCTGGAATAAGGCAAATGCTAAACGCCACAAA-3’ and 5’-TTTGTGGCGTTTAGCAGCTGCCGCATTCCAGGCCACCGG-3’. A NotI-SacII DNA fragment of BubR1 was used as a template.

For BubR1-E481K, mutagenizing primers were used to substitute a lysine for the glutamate 481 in the Bub3 binding domain of BubR1.

The amplified PCR fragment containing either the mutated KAN (AAA) or the mutated E481K was used to replace a corresponding NotI-SacII DNA fragment of the previously published RFP-BubR1 construct controlled by the natural bubR1 promoter (Buffin et al., 2005). All modifications were confirmed by sequencing. The constructs were introduced into the germ line of w1118 flies by standard methods using the helper plasmid (pUC-hs-Δ2-3). We obtained several independent transformants for RFP-BubR1-KAN (BubR1-KAN) and for RFP-BubR1-E481K (BubR1-E481K).

### Western blot

Proteins extracts from 5 brains of RFP-tagged BubR1 WT, BubR1-KEN1 or BubR1-KAN (each expressed in the *bubR1*^*1*^ mutant background) third instar larvae were loaded onto 8% SDS–acrylamide gels. Proteins were transferred to nitrocellulose membrane (Protran BA 85; Schleicher-Schuell) using an electrophoretic blotting device (Mini protean 3; Bio-Rad Laboratories). Membranes were blocked for 1 h in TBST (Tris-base 20 mM pH 7.5, 150 mM NaCl, 0.1% Tween) with 5% dry milk and incubated for 1.5 h with rabbit anti-RFP (Abcam) diluted at 1:1000 in TBST plus 1% milk. After washing in TBST, the blot was incubated for 1 h at room temperature with secondary antibody of goat anti–rabbit IgG conjugated with horseradish peroxidase (Promega) diluted at 1:2500. Immunodetection was performed with the SuperSignal kit (Thermo Fisher Scientific). Membranes were stripped and incubated with rabbit anti-Fzy diluted at 1:500 (gift from Jordan Raff, University of Oxford, Oxford, UK) followed by incubation with mouse anti–α tubulin (Sigma-Aldrich) diluted at 1:4000 to verify equal loading of proteins.

## References

[BIO021196C1] Basu J., Bousbaa H., Logarinho E., Li Z., Williams B. C., Lopes C., Sunkel C. E. and Goldberg M. L. (1999). Mutations in the essential spindle checkpoint gene bub1 cause chromosome missegregation and fail to block apoptosis in *Drosophila*. *J. Cell Biol.* 146, 13-28. 10.1083/jcb.146.999.13 10402457 PMC2199734

[BIO021196C2] Buffin E., Lefebvre C., Huang J., Gagou M. E. and Karess R. E. (2005). Recruitment of Mad2 to the kinetochore requires the Rod/Zw10 complex. *Curr. Biol.* 15, 856-861. 10.1016/j.cub.2005.03.052 15886105

[BIO021196C3] Buffin E., Emre D. and Karess R. E. (2007). Flies without a spindle checkpoint. *Nat. Cell Biol.* 9, 565-572. 10.1038/ncb1570 17417628

[BIO021196C4] Burton J. L. and Solomon M. J. (2007). Mad3p, a pseudosubstrate inhibitor of APCCdc20 in the spindle assembly checkpoint. *Genes Dev.* 21, 655-667. 10.1101/gad.1511107 17369399 PMC1820940

[BIO021196C5] Chao W. C. H., Kulkarni K., Zhang Z., Kong E. H. and Barford D. (2012). Structure of the mitotic checkpoint complex. *Nature* 484, 208-213. 10.1038/nature10896 22437499

[BIO021196C6] Derive N., Landmann C., Montembault E., Claverie M.-C., Pierre-Elies P., Goutte-Gattat D., Founounou N., McCusker D. and Royou A. (2015). Bub3-BubR1-dependent sequestration of Cdc20Fizzy at DNA breaks facilitates the correct segregation of broken chromosomes. *J. Cell Biol.* 211, 517-532. 10.1083/jcb.201504059 26553926 PMC4639866

[BIO021196C7] Di Fiore B., Davey N. E., Hagting A., Izawa D., Mansfeld J., Gibson T. J. and Pines J. (2015). The ABBA motif binds APC/C activators and is shared by APC/C substrates and regulators. *Dev. Cell* 32, 358-372. 10.1016/j.devcel.2015.01.003 25669885 PMC4713905

[BIO021196C8] Diaz-Martinez L. A., Tian W., Li B., Warrington R., Jia L., Brautigam C. A., Luo X. and Yu H. (2015). The Cdc20-binding Phe box of the spindle checkpoint protein BubR1 maintains the mitotic checkpoint complex during mitosis. *J. Biol. Chem.* 290, 2431-2443. 10.1074/jbc.M114.616490 25505175 PMC4303692

[BIO021196C9] Elowe S., Dulla K., Uldschmid A., Li X., Dou Z. and Nigg E. A. (2010). Uncoupling of the spindle-checkpoint and chromosome-congression functions of BubR1. *J. Cell Sci.* 123, 84-94. 10.1242/jcs.056507 20016069

[BIO021196C10] Emre D., Terracol R., Poncet A., Rahmani Z. and Karess R. E. (2011). A mitotic role for Mad1 beyond the spindle checkpoint. *J. Cell Sci.* 124, 1664-1671. 10.1242/jcs.081216 21511728

[BIO021196C11] Hardwick K. G., Johnston R. C., Smith D. L. and Murray A. W. (2000). MAD3 encodes a novel component of the spindle checkpoint which interacts with Bub3p, Cdc20p, and Mad2p. *J. Cell Biol.* 148, 871-882. 10.1083/jcb.148.5.871 10704439 PMC2174553

[BIO021196C12] Harris L., Davenport J., Neale G. and Goorha R. (2005). The mitotic checkpoint gene BubR1 has two distinct functions in mitosis. *Exp. Cell Res.* 308, 85-100. 10.1016/j.yexcr.2005.03.036 15907836

[BIO021196C13] Hewitt L., Tighe A., Santaguida S., White A. M., Jones C. D., Musacchio A., Green S. and Taylor S. S. (2010). Sustained Mps1 activity is required in mitosis to recruit O-Mad2 to the Mad1–C-Mad2 core complex. *J. Cell Biol.* 190, 25-34. 10.1083/jcb.201002133 20624899 PMC2911659

[BIO021196C14] Izawa D. and Pines J. (2015). The mitotic checkpoint complex binds a second CDC20 to inhibit active APC/C. *Nature* 517, 631-634. 10.1038/nature13911 25383541 PMC4312099

[BIO021196C15] Kiehart D. P., Montague R. A., Rickoll W. L., Foard D. and Thomas G. H. (1994). High-resolution microscopic methods for the analysis of cellular movements in *Drosophila* embryos. *Methods Cell Biol.* 44, 507-532. 10.1016/S0091-679X(08)60929-2 7707969

[BIO021196C16] King E. M. J., van der Sar S. J. A. and Hardwick K. G. (2007). Mad3 KEN boxes mediate both Cdc20 and Mad3 turnover, and are critical for the spindle checkpoint. *PLoS ONE* 2, e342 10.1371/journal.pone.0000342 17406666 PMC1829190

[BIO021196C17] Kulukian A., Han J. S. and Cleveland D. W. (2009). Unattached kinetochores catalyze production of an anaphase inhibitor that requires a Mad2 template to prime Cdc20 for BubR1 binding. *Dev. Cell* 16, 105-117. 10.1016/j.devcel.2008.11.005 19154722 PMC2655205

[BIO021196C18] Lara-Gonzalez P., Scott M. I. F., Diez M., Sen O. and Taylor S. S. (2011). BubR1 blocks substrate recruitment to the APC/C in a KEN-box-dependent manner. *J. Cell Sci.* 124, 4332-4345. 10.1242/jcs.094763 22193957 PMC3258114

[BIO021196C19] Larsen N. A., Al-Bassam J., Wei R. R. and Harrison S. C. (2007). Structural analysis of Bub3 interactions in the mitotic spindle checkpoint. *Proc. Natl. Acad. Sci. USA* 104, 1201-1206. 10.1073/pnas.0610358104 17227844 PMC1770893

[BIO021196C20] Li D., Morley G., Whitaker M. and Huang J.-Y. (2010). Recruitment of Cdc20 to the kinetochore requires BubR1 but not Mad2 in *Drosophila melanogaster*. *Mol. Cell. Biol.* 30, 3384-3395. 10.1128/MCB.00258-10 20421417 PMC2897573

[BIO021196C21] Lischetti T., Zhang G., Sedgwick G. G., Bolanos-Garcia V. M. and Nilsson J. (2014). The internal Cdc20 binding site in BubR1 facilitates both spindle assembly checkpoint signalling and silencing. *Nat. Commun.* 5, 5563 10.1038/ncomms6563 25482201

[BIO021196C22] Logarinho E., Bousbaa H., Dias J. M., Lopes C., Amorim I., Antunes-Martins A. and Sunkel C. E. (2004). Different spindle checkpoint proteins monitor microtubule attachment and tension at kinetochores in *Drosophila* cells. *J. Cell Sci.* 117, 1757-1771. 10.1242/jcs.01033 15075237

[BIO021196C23] Maciejowski J., George K. A., Terret M.-E., Zhang C., Shokat K. M. and Jallepalli P. V. (2010). Mps1 directs the assembly of Cdc20 inhibitory complexes during interphase and mitosis to control M phase timing and spindle checkpoint signaling. *J. Cell Biol.* 190, 89-100. 10.1083/jcb.201001050 20624902 PMC2911671

[BIO021196C24] Malureanu L. A., Jeganathan K. B., Hamada M., Wasilewski L., Davenport J. and van Deursen J. M. (2009). BubR1 N terminus acts as a soluble inhibitor of cyclin B degradation by APC/C(Cdc20) in interphase. *Dev. Cell* 16, 118-131. 10.1016/j.devcel.2008.11.004 19154723 PMC2659634

[BIO021196C25] Meraldi P., Draviam V. M. and Sorger P. K. (2004). Timing and checkpoints in the regulation of mitotic progression. *Dev. Cell* 7, 45-60. 10.1016/j.devcel.2004.06.006 15239953

[BIO021196C26] Musacchio A. and Salmon E. D. (2007). The spindle-assembly checkpoint in space and time. *Nat. Rev. Mol. Cell Biol.* 8, 379-393. 10.1038/nrm2163 17426725

[BIO021196C27] Raff J. W., Jeffers K. and Huang J.-Y. (2002). The roles of Fzy/Cdc20 and Fzr/Cdh1 in regulating the destruction of cyclin B in space and time. *J. Cell Biol.* 157, 1139-1149. 10.1083/jcb.200203035 12082076 PMC2173543

[BIO021196C28] Rahmani Z., Gagou M. E., Lefebvre C., Emre D. and Karess R. E. (2009). Separating the spindle, checkpoint, and timer functions of BubR1. *J. Cell Biol.* 187, 597-605. 10.1083/jcb.200905026 19951912 PMC2806589

[BIO021196C29] Rodriguez-Bravo V., Maciejowski J., Corona J., Buch H. K., Collin P., Kanemaki M. T., Shah J. V. and Jallepalli P. V. (2014). Nuclear pores protect genome integrity by assembling a premitotic and Mad1-dependent anaphase inhibitor. *Cell* 156, 1017-1031. 10.1016/j.cell.2014.01.010 24581499 PMC3947552

[BIO021196C30] Santaguida S., Tighe A., D'Alise A. M., Taylor S. S. and Musacchio A. (2010). Dissecting the role of MPS1 in chromosome biorientation and the spindle checkpoint through the small molecule inhibitor reversine. *J. Cell Biol.* 190, 73-87. 10.1083/jcb.201001036 20624901 PMC2911657

[BIO021196C31] Schittenhelm R. B., Heeger S., Althoff F., Walter A., Heidmann S., Mechtler K. and Lehner C. F. (2007). Spatial organization of a ubiquitous eukaryotic kinetochore protein network in *Drosophila* chromosomes. *Chromosoma* 116, 385-402. 10.1007/s00412-007-0103-y 17333235 PMC1950589

[BIO021196C32] Sczaniecka M., Feoktistova A., May K. M., Chen J.-S., Blyth J., Gould K. L. and Hardwick K. G. (2008). The spindle checkpoint functions of Mad3 and Mad2 depend on a Mad3 KEN box-mediated interaction with Cdc20-anaphase-promoting complex (APC/C). *J. Biol. Chem.* 283, 23039-23047. 10.1074/jbc.M803594200 18556659 PMC2516979

[BIO021196C33] Sudakin V., Chan G. K. T. and Yen T. J. (2001). Checkpoint inhibition of the APC/C in HeLa cells is mediated by a complex of BUBR1, BUB3, CDC20, and MAD2. *J. Cell Biol.* 154, 925-936. 10.1083/jcb.200102093 11535616 PMC2196190

[BIO021196C34] Tang Z., Bharadwaj R., Li B. and Yu H. (2001). Mad2-Independent inhibition of APCCdc20 by the mitotic checkpoint protein BubR1. *Dev. Cell* 1, 227-237. 10.1016/S1534-5807(01)00019-3 11702782

[BIO021196C35] Tian W., Li B., Warrington R., Tomchick D. R., Yu H. and Luo X. (2012). Structural analysis of human Cdc20 supports multisite degron recognition by APC/C. *Proc. Natl. Acad. Sci. USA* 109, 18419-18424. 10.1073/pnas.1213438109 23091007 PMC3494910

[BIO021196C36] Vleugel M., Hoogendoorn E., Snel B. and Kops G. J. P. L. (2012). Evolution and function of the mitotic checkpoint. *Dev. Cell* 23, 239-250. 10.1016/j.devcel.2012.06.013 22898774

[BIO021196C37] Williams B. C., Li Z., Liu S., Williams E. V., Leung G., Yen T. J. and Goldberg M. L. (2003). Zwilch, a new component of the ZW10/ROD complex required for kinetochore functions. *Mol. Biol. Cell* 14, 1379-1391. 10.1091/mbc.E02-09-0624 12686595 PMC153108

